# Opposing alterations in anxiety and species-typical behaviours in serotonin transporter overexpressor and knockout mice

**DOI:** 10.1016/j.euroneuro.2010.08.005

**Published:** 2011-01

**Authors:** Samantha J. Line, Christopher Barkus, Clare Coyle, Katie A. Jennings, Robert M. Deacon, Klaus P. Lesch, Trevor Sharp, David M. Bannerman

**Affiliations:** a Department of Experimental Psychology, South Parks Road, Oxford, OX1 3UD, United Kingdom; b Department of Pharmacology, Mansfield Road, Oxford, OX1 3QT, United Kingdom; c ADHD Clinical Research Network, Unit for Molecular Psychiatry, Laboratory of Translational Neuroscience, Department of Psychiatry, Psychosomatics and Psychotherapy, University of Wuerzburg, Wuerzburg, Germany

**Keywords:** 5-HT;, 5-HT transporter;, Anxiety;, Transgenic mice;, Body weight

## Abstract

Human gene association studies have produced conflicting findings regarding the relationship between the 5-HT transporter (5-HTT) and anxiety. In the present study genetically modified mice were utilised to examine the effects of changes in 5-HTT expression on anxiety. In addition, the influence of 5-HTT expression on two innate “species-typical” behaviours (burrowing and marble burying) and body weight was explored. Across a range of models, 5-HTT overexpressing mice displayed reduced anxiety-like behaviour whilst 5-HTT knockout mice showed increased anxiety-like behaviour, compared to wildtype controls. In tests of species-typical behaviour 5-HTT overexpressing mice showed some facilitation whilst 5-HTT knockout mice were impaired. Reciprocal effects were also seen on body weight, as 5-HTT overexpressors were lighter and 5-HTT knockouts were heavier than wildtype controls. These findings show that variation in 5-HTT gene expression produces robust changes in anxiety and species-typical behaviour. Furthermore, the data add further support to findings that variation of 5-HTT expression in the human population is linked to changes in anxiety-related personality traits.

## 1 Introduction

The neurotransmitter 5-hydroxytryptamine (5-HT/serotonin) has been linked to the pathology of a number of psychiatric disorders, including depression, anxiety, obsessive compulsive disorder and eating disorders (Owens and Nemeroff, 1994; Handley, 1995; Gorwood, 2004; Hu et al., 2006). After release, 5-HT is removed from the synapse primarily by the 5-HT transporter (5-HTT), which is thus instrumental in regulating 5-HT activity (Bradley and Blakely, 1997). Studies have demonstrated large natural variation in 5-HTT expression, which is likely to be influenced by both genetic and environmental factors (Pirker et al., 2000; Rhodes et al., 2007). However, the effects of this variation on personality, cognition, and psychopathology are poorly understood.

The 5-HTT-linked promoter region (5-HTTLPR) contains a 44 base pair insertion/deletion polymorphism which gives rise to a long (*l*) or a short (*s*) allele (Heils et al., 1996) with the *s* allele leading to reduced 5-HTT expression and the *l* allele increasing 5-HTT expression (Heils et al., 1996; Lesch et al., 1996; Hu et al., 2006), although this remains controversial (Mann et al., 2000; Preuss et al., 2000; Parsey et al., 2006). The *s* allele has been associated with a number of outcomes, including anxiety-related personality traits (Lesch et al., 1996; Du et al., 2000; Greenberg et al., 2000; Melke et al., 2001), mood disorders (Lotrich and Pollock, 2004; Lasky-Su et al., 2005), and suicide (Anguelova et al., 2003; Roy et al., 2007). *s* allele carriers have also been found to display significantly greater amygdala activation to fearful faces (Hariri et al., 2002; Hariri et al., 2005) aversive pictures (Heinz et al., 2005) and negative words (Canli et al., 2005) compared to non-carriers, which may indicate a role for amygdala hyperresponsivity in the observed vulnerabilities. In addition, *s* allele carriers appear to be more sensitive to stressful life events (Caspi et al., 2003; Pluess et al., 2010).

A major difficulty with these studies is that the multitude of genetic and environmental factors which influence behaviour in heterogeneous human populations makes it difficult to firmly establish the role of single genes. Because of this, genetic mouse models have been developed to examine the effect of changes in the expression of the 5-HTT in isolation from other influences. Initial studies examined the effects of loss-of-function of the 5-HTT and observed increased anxiety in some circumstances (Holmes et al., 2001; Holmes et al., 2003a; Holmes et al., 2003b). However, although the 5-HTT knockout (KO) mouse provides useful clues as to the role of the 5-HTT, complete loss-of-function of the 5-HTT is not observed in humans. Thus, an overexpressor (OE) mouse was developed with 5-HTT expression increased to levels similar to those expected from the high expressing human 5-HTT gene variants (Heils et al., 1996; Lesch et al., 1996; Jennings et al., 2006). Furthermore, in comparison to the effects of 5-HTT KO, an initial study indicated reduced anxiety in these animals (Jennings et al., 2006).

Here we aimed to compare 5-HTT KO and 5-HTT OE mice with respective wildtype controls on a range of anxiety tasks with varying sensorimotor and motivational demands. In addition, the performance of these mice in three measures of “species-typical” behaviour was investigated. Although previous findings have suggested impaired species-typical behaviour in 5-HTT KO mice (Zhao et al., 2006), 5-HTT OE mice have not been examined. This is significant as these behaviours are sensitive to pharmacological blockade of the 5-HTT (Njung'e and Handley, 1991; Ichimaru et al., 1995).

## 2 Experimental procedures

For full methods please see supporting supplementary information.

### 2.1 Animals

Experiments were conducted in accordance with the United Kingdom Animals (Scientific Procedures) Act of 1986. 5-HTT OE mice and wildtype (WT) littermates were generated on a CBA x C57BL/6J background, as described previously (Jennings et al., 2006), and bred in the University of Oxford. 5-HTT KO mice and WT littermates were generated on a 129P1 (129P1/ReJ) x C57BL/6J hybrid background, before being repeatedly backcrossed onto a C57BL/6J background for more than eight generations (Bengel et al., 1998). Both males and females were examined on all tasks. Mice were group housed (4–6 per cage) and all animals were provided with enrichment and *ad libitum* food and water unless otherwise stated. Mice were maintained on a 12 h light/dark cycle (lights off 19:00 to 7:00) in a temperature-controlled environment (21 ± 1 °C). Three separate cohorts were used for the tests of anxiety, locomotor activity and species-typical behaviour.

### 2.2 Behavioural protocols

Tasks were performed in the order described with no more than one task performed per day.

#### 2.2.1 Anxiety tasks

##### 2.2.1.1 Elevated plus maze

The plus maze consisted of two “open” arms and two “closed” arms, arranged in a plus formation, joined by a central rectangular region Animals were placed individually at the distal end of a closed arm facing away from the centre, and were allowed to explore the apparatus for 300 s. The amount of time spent in the open arms, number of entries into the open arms, total number of arm entries, and latency to first enter an open arm were measured.

##### 2.2.1.2 Hyponeophagia

Prior to testing animals were food deprived overnight for approximately 18 h. Animals were presented with a novel food in an unfamiliar environment and the latency to begin continuous eating was recorded.

##### 2.2.1.3 Successive alleys

The apparatus consisted of four successive, increasingly anxiogenic (each succeeding alley was painted a lighter colour, had lower walls and/or was narrower than the previous alley) linearly connected alleys. Animals were placed at the closed end of alley 1, facing the end wall. The latency to first enter each alley, the amount of time spent in each alley, and the number of entries into each alley were recorded during a total test time of 300 s.

##### 2.2.1.4 Black-white alley

The test apparatus consisted of a long alley with the floor and walls of one half painted black and the other half painted white. Each mouse was placed individually into the black section of the test alley facing the end wall. The mouse was observed for 120 s. Latency to first cross from the black section to the white section, total time spent in the white section, and number of crossings between the two sections were recorded.

#### 2.2.2 Locomotor activity

Spontaneous locomotor activity was assessed by placing mice individually in transparent plastic cages. Two infrared beams crossed each cage and the number of beam breaks made per 5 min time bin was recorded during a 2 h test session.

#### 2.2.3 Species-typical behaviour

##### 2.2.3.1 Burrowing

Burrows consisted of grey plastic cylinders raised at the open end and filled with 200 g of standard laboratory chow. Each animal was left undisturbed with a burrow for 2 h, after which the amount burrowed was recorded (Deacon, 2006).

##### 2.2.3.2 Marble burying

Transparent plastic cages were filled with a 10 cm deep layer of sawdust on top of which 10 glass marbles were placed in two rows. Each animal was left undisturbed in a cage for 30 min, after which the number of marbles that were buried to at least 2/3 of their depth was recorded.

### 2.3 Statistical analysis

Data from the two cohorts (5-HTT OEs and KOs) were analysed separately. Parametric data were analysed using two-way ANOVAs (genotype and sex as between-subject factors). Where data violated assumptions of normality or equality of variance, transformations (log10 or square root) were utilised. For repeated measures ANOVAs, homogeneity of variance was tested using Mauchly's test of sphericity, and where this was violated, Huyn–Feldt corrections were used. Non-parametric data were analysed using Mann–Whitney U tests. A p-value < 0.05 was considered statistically significant throughout.

## 3 Results

### 3.1 Anxiety tests

#### 3.1.1 Elevated plus maze

**Figure 1:**
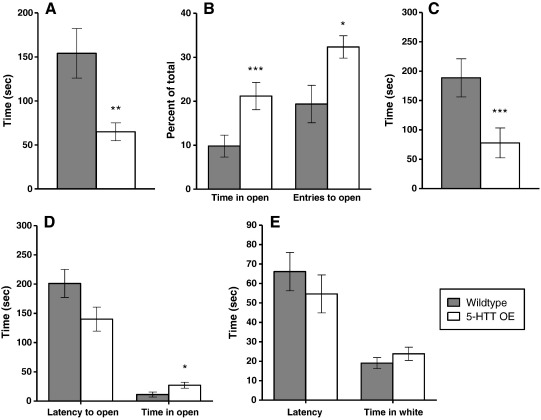
5-HTT OE mice on anxiety tasks. A: Elevated plus maze — latency to enter an open arm (WT n = 19; OE n = 19). B: Elevated plus maze — percentage time spent in open arms and percentage of entries to open arms (WT n = 19; OE n = 19). C: Hyponeophagia — latency to begin eating (WT n = 21; OE n = 22). D: Successive alleys — latency to enter the first open alley and time spent in the open alleys (WT n = 23; OE n = 25). E: Black-white alley — latency to enter white alley and time in white alley (WT n = 23; OE n = 25). Values represent the mean ± SEM. *p < 0.05; **p < 0.01; ***p < 0.005 compared to wildtype littermates.

**Figure 2:**
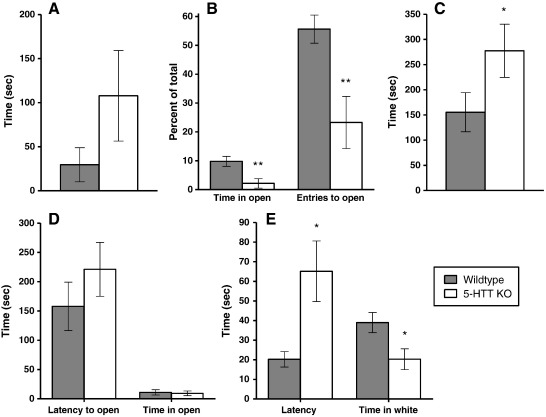
5-HTT KO mice on anxiety tasks. A: Elevated plus maze — latency to enter an open arm (WT n = 9; KO n = 7). B: Elevated plus maze — percentage time spent in open arms and percentage of entries to open arms (WT n = 9; KO n = 7). C: Hyponeophagia — latency to begin eating (WT n = 9; KO n = 7). D: Successive alleys — latency to enter the first open alley and time spent in open alleys (WT n = 9; KO n = 7). E: Black-white alley — latency to enter white alley and time in white alley (WT n = 9; KO n = 7). Values represent the mean ± SEM. *p < 0.05; **p < 0.01 compared to wildtype littermates.

5-HTT OE mice were significantly faster to enter an open arm [F(1,34) = 9.17 p < 0.01] (Fig. 1A), spent a significantly greater proportion of time in the open arms [F(1,34) = 10.16 p < 0.005] (Fig. 1B) and made a greater proportion of entries into the open arms [F(1,34) = 6.42 p < 0.05] than their wildtype littermates. In contrast, although 5-HTT KO mice did not differ from wildtypes on the latency to first enter an open arm [F(1,12) = 2.20 p = 0.16] (Fig. 2A), they spent a significantly reduced proportion of time in the open arms [F(1,12) = 11.64 p < 0.01], and made a significantly smaller proportion of entries into these arms [F(1,12) = 15.23 p < 0.005] (Fig. 2B) compared to wildtypes. Neither the 5-HTT OE mice [F(1,34) = 2.73 p = 0.11], nor the 5-HTT KO mice [F(1,12) = 1.59 p = 0.23], differed from their wildtype littermates on the number of entries made into the closed arms of the maze, suggesting that differences in open arm behaviour do not simply reflect general changes in locomotor activity. There were no significant effects of sex or genotype * sex interactions on any measure.

#### 3.1.2 Hyponeophagia

When presented with a novel food in a novel environment, 5-HTT OE mice did not differ from wildtypes on the latency to make first contact with the food [F(1,39) = 1.12 p = 0.30] (data not shown), but were significantly faster to begin eating [F(1,39) = 11.32 p < 0.005] (Fig. 1C). Although males had a significantly longer latency to begin eating than females [F(1,39) = 4.22 p < 0.05], there was no significant genotype * sex interaction. In contrast to 5-HTT OE mice, 5-HTT KO mice took significantly longer to begin eating than their wildtype littermates [F(1,12) = 4.78 p < 0.05] (Fig. 2C). There were no significant effects of sex or a genotype * sex interaction.

#### 3.1.3 Successive alleys

Because only a very small number of animals explored further than alley 2, the results from alleys 2 to 4 (the “open” alleys) were combined. The 5-HTT OE mice displayed a significantly shorter latency to enter the first open alley [F(1,44) = 4.08 p = 0.05] (Fig. 1D) and spent significantly more time in the open alleys [F(1,44) = 5.62 p < 0.05] than wildtypes. They also made more crossings between alleys [F(1,44) = 6.71 p < 0.05]. In the 5-HTT KO cohort no significant effects of genotype were present on any measure [F < 1] (Fig. 2D). There were no significant effects of sex or genotype * sex interactions in either cohort.

#### 3.1.4 Black-white alley

5-HTT OE mice did not differ from wildtypes on either the latency to enter the white half of the alley [F(1,44) = 1.80 p = 0.19] (Fig. 1E) or the total amount of time spent in this region [F(1,44) = 1.04 p = 0.31]. However, 5-HTT KO mice were significantly slower to enter the white part of the alley [F(1,12) = 8.93 p < 0.05] (Fig. 2E) and spent significantly less time in this region [F(1,12) = 5.36 p < 0.05] compared to wildtypes. Neither the 5-HTT OE mice nor the 5-HTT KO mice differed from their wildtype littermates on the number of crossings made between the two regions ([F < 1] and [F(1,12) = 2.48 p < 0.14], respectively). Although female 5-HTT KO mice made significantly fewer crossings than male 5-HTT KO mice [F(1,12) = 5.10 p < 0.05], no other effects of sex or genotype * sex interactions were present.

### 3.2 Locomotor activity

**Figure 3:**
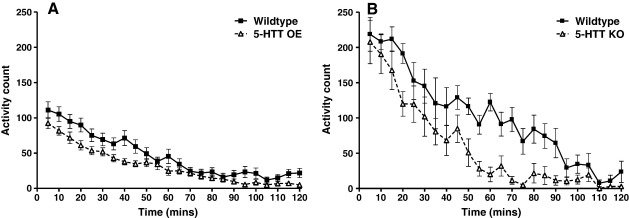
Locomotor activity in 5-HTT OE and KO mice compared to their respective wildtype littermates. A: Locomotor activity in 5-HTT OE mice and wildtype littermates (WT n = 23; OE n = 25). B: Locomotor activity in 5-HTT KO mice and wildtype littermates (WT n = 9; KO n = 7). Values represent mean photocell beam breaks per 5 min block ± SEM.

Both 5-HTT OE and 5-HTT KO mice exhibited significantly less spontaneous locomotor activity than their wildtype littermates ([F(1,44) = 7.27 p < 0.05] (Fig. 3A) and [F(1,12) = 13.60 p < 0.005] (Fig. 3B), respectively). Although males were more active than females in the KO cohort [F(1,12) = 10.68 p < 0.01], there were no genotype * sex interactions in either cohort.

### 3.3 Species-typical behaviour

#### 3.3.1 Burrowing

**Figure 4:**
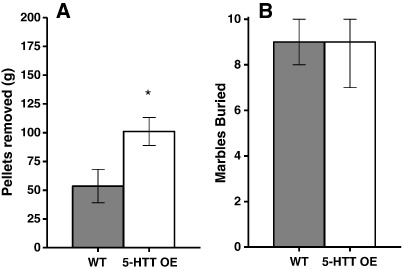
Species-typical behaviour in 5-HTT OE mice. A: Weight of pellets burrowed (mean ± SEM). B: Marbles buried out of 10 (median ± IQR). WT n = 21; OE n = 29. *p < 0.05 compared to wildtype littermates.

**Figure 5:**
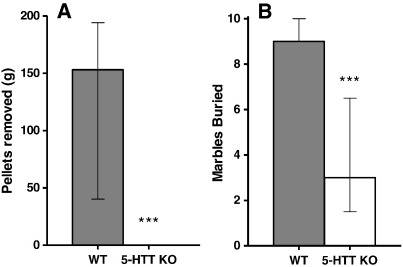
Species-typical behaviour in 5-HTT KO mice. A: Weight of pellets burrowed (median ± IQR). B: Marbles buried out of 10 (median ± IQR). WT n = 8; KO n = 7. ***p < 0.005 compared to wildtype littermates.

5-HTT OE mice burrowed a significantly greater weight of pellets than wildtypes [F(1,37) = 4.96 p < 0.05] (Fig. 4A). In contrast, 5-HTT KO mice failed to burrow [U(7,8) = 52.5 p < 0.005] (Fig. 5A). There were no significant effects of sex.

#### 3.3.2 Marble burying

The number of marbles buried by the 5-HTT OE mice did not differ from the number buried by their wildtype littermates [U(21,29) = 270 p = 0.48] (Fig. 4B). However, 5-HTT KO mice buried significantly fewer marbles than wildtypes [U(7,8) = 55.5 p < 0.001] (Fig. 5B).

### 3.4 Body weight

Mice were weighed at approximately 6 months of age. 5-HTT OE mice were significantly lighter than their wildtype littermates (wildtype = 31.4 g ± 0.9 OE = 24.5 g ± 0.6 [F(1,42) = 56.14 p < 0.001]) whilst 5-HTT KO mice were significantly heavier than wildtypes (wildtype = 23.6 g ± 1.4 KO = 30.4.5 g ± 3.5 [F(1,12) = 7.23 p < 0.05]). Males were heavier than females in both cohorts ([F(1,42) = 8.07 p < 0.01] and [F(1,12) = 24.87 p < 0.001], respectively). In addition, there was a trend towards a genotype * sex interaction in the 5-HTT OE mice [F(1,42) = 3.68 p = 0.06] because the genetic manipulation had a greater effect on males than females, and a significant genotype * sex interaction in the 5-HTT KO mice [F(1,12) = 5.48 p < 0.05], because loss of the 5-HTT only had an effect on the body weight of males.

## 4 Discussion

The results of these studies demonstrate a powerful impact of variation in 5-HTT expression on anxiety-related behaviour. Thus, across a range of tasks, genetically-manipulated mice with increased 5-HTT expression showed reduced anxiety-like behaviour, whereas mice lacking 5-HTT expression showed increased anxiety. The results also indicate the importance of 5-HTT expression in species-typical behaviour, with increased and decreased 5-HTT expression being associated with facilitation and disruption, respectively. These differences were observed in both male and female mice, suggesting that the effects of altered 5-HTT expression on anxiety are not influenced by sex in these tests.

### 4.1 Anxiety

5-HTT OE mice displayed reduced anxiety on the elevated plus maze, hyponeophagia and successive alleys tasks, whilst 5-HTT KO animals showed increased anxiety on the elevated plus maze, hyponeophagia and black-white alleys tasks. The finding that differences were not observed in both strains on all tasks may result from floor/ceiling effects (e.g. 5-HTT KO on the successive alleys). However, the present data suggest that both lines exhibit robust changes in anxiety. In particular, the findings of altered anxiety in both (i) the hyponeophagia test and (ii) the elevated plus maze/successive alleys tests suggest that the observed anxiety phenotypes are unlikely to be due to non-specific alterations in motivation or locomotion as these tests produced corresponding results despite differing sensorimotor and motivational demands.

Anxiety can be conceptualised as occurring when an animal experiences conflict between approach and avoidance responses (Gray and McNaughton, 2000). As such, increased anxiety manifests as a greater degree of behavioural inhibition, resulting in increased threat avoidance. Therefore, changes in baseline locomotor activity can present a difficulty when interpreting the results of anxiety tasks as they can both result from, and give the appearance of, alterations in anxiety. For example, reduced locomotor activity in the 5-HTT KO mice might result in reduced open arm entries on the plus maze and thus cause them to appear anxious. However, the lack of a change in closed arm entries suggests that the change in open arm behaviour cannot be explained solely by altered locomotor activity. In addition, the 5-HTT KO animals were significantly slower to begin eating on the test of hyponeophagia, suggesting increased anxiety independent of locomotor activity (Britton and Britton, 1981; Shephard and Broadhurst, 1982; Shephard et al., 1984). Changes in baseline locomotor activity are also unable to explain the reduction in anxiety-like behaviour of the 5-HTT OE mice as the reduced spontaneous activity seen in these animals would be expected to make them appear more – not less – anxious on tests such as the elevated plus maze, successive alleys and black-white alley. Therefore, it is unlikely that differences in baseline locomotor activity account for the observed relationship between anxiety and 5-HTT expression.

The behaviour of the two groups of wildtypes differed considerably, which is likely to result from the different genetic background of the two lines. The 5-HTT KO mutation was backcrossed onto a C57/BL6 background whilst the 5-HTT OE mutation remained on a hybrid background, which would be expected to increase intra-group variation in these animals. However, because transgenic animals were compared only to their respective wildtype littermates, and because the observed phenotypes were diametrically opposite, the differing background strains of the two cohorts are unlikely to account for the pattern of results seen.

The observed anxiety phenotypes are in agreement with the putative effects of the 5-HTTLPR on 5-HTT expression and anxiety traits in humans. In studies of human personality, the *l* allele of the 5-HTT has been associated with lower levels of neuroticism and associated anxiety-related traits (Lesch et al., 1996; Du et al., 2000; Greenberg et al., 2000; Melke et al., 2001). Although not all studies have concurred, there is evidence that individuals carrying two copies of the *l* allele (in particular, the *l*_*A*_ allele) of the 5-HTTLPR exhibit 2–3 times higher levels of 5-HTT expression and/or uptake activity (Heils et al., 1996; Lesch et al., 1996; Hanna et al., 1998; Greenberg et al., 1999; Heinz et al., 2000; Hu et al., 2006). This increase is comparable in magnitude to that seen in 5-HTT OE mice (both approximately 1.5 to 3-fold) (Jennings et al., 2006). Thus, the current findings suggest a direct link between increased 5-HTT expression and reduced anxiety, and that decreased levels of anxiety-related traits observed in *l* allele homozygotes may be the result of higher levels of expression of the 5-HTT. In contrast, selective serotonin reuptake inhibitors (SSRIs) act to reduce the activity of the 5-HTT and yet have anxiolytic effects (Katzman, 2009). This suggests that genetic alterations in 5-HTT expression (which are present throughout development) result in qualitatively different effects on anxiety to those produced by pharmacological manipulations during adulthood. The causes of this are likely to be complex, but may reflect developmental alterations in stress reactivity (Mueller et al., 2010; Way and Taylor, 2010).

Previous work suggests that 5-HTT OE mice display a normal distribution of 5-HTT expression, which is largely confined to raphé 5-HT neurons and their projections (Jennings et al., 2006). However, because these projections are spread across a variety of cortical and subcortical regions, changes in 5-HTT expression results in altered 5-HT activity in a number of brain regions. This makes it difficult to draw firm conclusions regarding the anatomical basis of the phenotypes displayed by 5-HTT OE and KO animals. Despite this, it is notable that all the anxiety tasks described here are sensitive to lesions of the ventral hippocampus (Bannerman et al., 2003; Bannerman et al., 2004; McHugh et al., 2004), whereas they are not affected by amygdala lesions (Treit et al., 1993; Kjelstrup et al., 2002; McHugh et al., 2004). Thus these data – coupled with the known serotonergic innervation of the hippocampus (McQuade and Sharp, 1997; Adams et al., 2008) – implicate the hippocampal formation as a possible anatomical substrate of some of the altered behaviours seen in 5-HTT mutants.

### 4.2 Species-typical behaviour

5-HTT KO mice have previously been found to display reduced marble burying behaviour (Zhao et al., 2006), which was confirmed by the present study, whereas 5-HTT OE mice did not differ from wildtypes on this test (although this may have been due to ceiling effects). Exploration of species-typical behaviours was expanded with an examination of burrowing. Although burrowing has been utilised less than marble burying in the laboratory, it represents a sensitive and robust test of species-typical behaviour and – like marble burying – has been shown to be hippocampal-dependent (Deacon et al., 2002; Deacon, 2006). 5-HTT KO mice displayed a dramatic impairment in burrowing (none of these mice performed any burrowing), whilst 5-HTT OE animals showed a significant increase in this behaviour. This indicates that – as with anxiety-like behaviour – opposing alterations in 5-HTT expression produce opposite effects on species-typical behaviour.

### 4.3 Body weight

5-HT is known to have a suppressive effect on feeding through its actions in both the central nervous system and periphery (Blundell, 1986; Silverstone and Goodall, 1986; Leibowitz et al., 1988; Neill and Cooper, 1989) and drugs which act on the 5-HTT to increase 5-HT availability (such as fenfluramine) have been used clinically to induce weight-loss (Davis and Faulds, 1996; Carvajal et al., 2000). However – as was seen with anxiety – the effects of genetically-induced changes in 5-HTT activity on body weight were diametrically opposed to the effects of acute pharmacological manipulations. Thus, 5-HTT overexpression caused a significant reduction in body weight compared to wildtypes, whereas 5-HTT knockout resulted in an increase in body weight. These results also concord with studies associating the *s* allele of the 5-HTTLPR with obesity (Sookoian et al., 2007; Sookoian et al., 2008; Lan et al., 2009). As these animals have previously been found to display normal feeding behaviour (Pringle et al., 2008), 5-HTT overexpression may result in peripheral metabolic or developmental changes. It is also interesting to note that there are indications that the genetic manipulation had a larger effect on the body weight of males than females. This is in agreement with previous findings in animals (Uceyler et al., 2010), and raises the possibility that the 5-HTTLPR might have a greater influence on obesity in males than females.

### 4.4 Conclusions

Overall, these findings provide direct evidence that changes in 5-HTT gene expression have robust effects on anxiety and species-typical behaviour. In addition, the findings of this study correlate well with what is known of the behavioural effects of 5-HTT gene variants in humans, supporting the hypothesis that these phenotypes are the result of altered 5-HTT expression.

The following are the supplementary materials related to this article.

Supplementary Material

## Role of the funding source

Funding for this study was provided by a Wellcome Trust studentship awarded to Samantha Line and a grant from NEWMOOD awarded to Trevor Sharp. The funding bodies had no further role in study design; in the collection, analysis and interpretation of data; in the writing of the report; and in the decision to submit the paper for publication.

## Contributors

S. Line performed most of the experimental work and prepared the first draft of the manuscript. C. Barkus performed some of the experimental studies of species-typical behaviour and assisted in the preparation of the manuscript. C. Coyle performed some of the experimental studies of the 5-HTT KO mice. K. Jennings genotyped the animals and maintained the mouse colonies. R. Deacon designed and assisted in the tests of species-typical behaviour. K. Lesch provided the 5-HTT KO mice. T. Sharp advised on experimental design and assisted in the preparation of the manuscript. D. Bannerman advised on experimental design, interpretation and statistical analysis and assisted in the preparation of the manuscript.

## Conflict of interest

C. Barkus's PhD studentship is funded by GSK, but none of the studies described in this manuscript were carried out as part of his doctoral work.
