# Supplementary material for: Opposing alterations in anxiety and species-typical behaviours in serotonin transporter overexpressor and knockout mice
Source: Eur Neuropsychopharmacol. 2011 Jan;21(1):108–16. doi: 10.1016/j.euroneuro.2010.08.005 (PMC3038260; doi:10.1016/j.euroneuro.2010.08.005)
Supplement: Supplementary Material [file mmc1.doc]

## Supplementary Material

## Methods

### Animals

Experiments were conducted in accordance with the United Kingdom Animals (Scientific Procedures) Act of 1986. Both males and females were examined on all tasks. Mice were group housed (4-6 per cage) and all animals were provided with enrichment and *ad libitum* food and water unless otherwise stated. Mice were maintained on a 12 h light/dark cycle (lights off 19:00 to 7:00) in a temperature-controlled environment (21 ± 1°C).

#### Generation and Breeding of 5-HTT OE Mice

Mice were generated as described by Jennings et al. (2006). In summary: A 500 kb yeast artificial chromosome (YAC) containing the human 5-HTT gene with the "short" allele of the 5-HTTLPR in the promoter region and the 10-repeat allele of the variable number tandem repeat in intron 2 was constructed (Shen et al. 2000). YAC DNA was injected into fertilized eggs (CBA x C57BL/6J) and transgenic mice were identified by PCR using primer pairs for the human 5-HTT (exon 1A, exon 1B, intron 1A, and 3' untranslated region) and the YAC vector arms (Shen et al., 2000a). The mutation was maintained on a hybrid background through the repeated breeding of transgenic males with wildtype CBA x C57BL/6J F1 females.

#### Generation and Breeding of 5-HTT KO Mice

Mice were generated as described by Bengel et al. (1998). In summary: 3′ and 5′ DNA fragments encompassing exon 2 of the mouse 5-HTT were inserted into a pPNT-neo replacement targeting vector. A 1.1 kb fragment containing 5-HTT exon 2 was replaced by a 1.8 kb PGK neomycin-polyA expression cassette. The targeting construct was electroporated into 129 R1 embryonic stem cells and recombinant cell clones were identified by Southern blot analysis. Recombinant stem cell clones were then microinjected in C57BL/6J blastocysts to obtain chimeric progeny. Chimeric males were mated to C57BL/6J female mice and after confirmation of germline transmission, were repeatedly backcrossed onto a C57BL/6J background for more than eight generations (Bengel et al. 1998). 5-HTT+/−mice were mated to produce 5-HTT−/− mutants.

### Behavioural Protocols

##### Anxiety Tasks

A variety of anxiety tests with differing motivational and locomotor components were employed in order to limit the influence of potential confounding factors.

#### Elevated Plus Maze

The elevated plus maze draws on the conflict between the drive to explore new environments versus the drive to avoid danger. A highly anxious animal will spend less time in the open, exposed arms of the plus maze and more time in the closed, sheltered arms. In a less anxious animal the reverse will be true. Correspondingly, anxiolytic drugs (e.g. benzodiazepines) increase the amount of time animals spend in the open arms, whilst anxiogenic drugs (e.g. caffeine, picrotoxin) have the opposite effect (Handley and Mithani 1984; Lister 1987). Hippocampal lesions also increase the proportion of time animals spend on the open arms (Bannerman et al. 2002)

The plus maze consisted of two “open” arms and two “closed” arms, arranged in a plus formation, joined by a central rectangular region of 8 x 4 cm. The open arms were 29 x 4 cm and the closed arms were 27 x 8 cm, surrounded by 30 cm high clear Perspex walls. The arms were composed of white plastic, and the maze was elevated 50 cm above the ground. The room was illuminated by a 60 W bulb in a standard lamp directed away from the apparatus. Animals were placed individually at the distal end of a closed arm facing away from the centre, and were allowed to explore the apparatus for 300 sec. The amount of time spent in the open arms, number of entries into the open arms, total number of arm entries, and latency to first enter an open arm were measured.

#### Hyponeophagia

When animals are presented with a novel food in an unfamiliar environment the latency to begin consuming the food provides a measure of anxiety. The hyponeophagia test (also referred to as "novelty suppressed feeding") evaluates the latency of a rodent to begin eating a novel foodstuff in an unfamiliar environment. Depending on the baseline anxiety/neophobia of the animals being tested, the testing conditions can be varied in order to avoid floor or ceiling effects. Ultimately the latency to begin eating measures the outcome of conflicting drives in the animal (the drive to obtain food versus the drive to avoid unknown situations that may be dangerous) and the balance of these depends on baseline anxiety. Manipulations which are known to increase anxiety have been found to increase the latency to eat, while anxiolytic manipulations decrease it (Britton and Britton 1981; Shephard and Broadhurst 1982; Shephard et al. 1984; Merali et al. 2003). Lesions of the ventral hippocampus have also been found to reduce the latency to begin eating (Bannerman et al. 2002; Bannerman et al. 2003; McHugh et al. 2004).

Prior to testing animals were food deprived overnight for approximately 18 hours in order to increase their motivation to eat. 5-HTT OE mice and their wildtype littermates were placed individually on an unpainted wooden platform (24 x 11.5 cm, surrounded by a 1 cm wall), facing away from a small metal food well (12mm diameter) containing a 45mg sucrose pellet (Noyes). The latencies to i) first make contact with the pellet and ii) initiate continuous eating were recorded. Because of the high level of anxiety in the 5-HTT KO mice a less anxiogenic testing environment was utilised for these animals (and wildtype littermates) in order to facilitate eating. These animals were placed directly on a layer of food pellets (Noyes) in a small Tupperware container. The latency to initiate continuous eating was recorded.

For both tests, if eating was not initiated after 120 sec the animal was removed from the apparatus and placed in a homecage-like box for approximately 2 minutes. If after three separate 120 sec test sessions it still had not eaten, then the experiment was ended and a latency of 360 sec was recorded.

#### Successive Alleys

The successive alleys test is a modified form of the elevated plus maze consisting of four successive, increasingly anxiogenic, linearly connected alleys. Anxiety is assessed by observing how many alleys an animal progresses through, and how much time it spends on each of the alleys. Thus, the successive alleys task potentially offers a greater range of anxiogenic conditions than the plus maze whilst avoiding the ambiguities of a central region. Anxiolytic benzodiazepines and lesions of the ventral hippocampus increase the time animals spend on the distal open arms (McHugh et al. 2004).

Each of the four alleys was 25 cm long and made of painted wood. Alley 1 (the “closed alley”) was painted black, had 25 cm walls and was 8.5 cm wide. Alley 2 was painted grey and attached to alley 1 with a 0.5 cm step down. Alley 2 was also 8.5 cm wide, but had 1.3 cm walls. A 1.0 cm step down led to alley 3, which was 3.5 cm wide, had 0.8 cm walls, and was painted white. A further 0.4 cm step down led to alley 4, which was 1.2 cm wide with 0.2 cm walls, and was also painted white. The apparatus was elevated approximately 50 cm above the floor. Because alleys 2, 3 and 4 had very short walls these were termed “open alleys”. Animals were placed at the closed end of alley 1, facing the end wall. The latency to first enter each alley, the amount of time spent in each alley, and the number of entries into each alley were recorded during a total test time of 300 sec. If the animals had not entered the second alley by the end of the test, they were assigned a latency of 300 sec.

#### Black-White Alley

The black-white alley also exploits the approach/avoidance conflict between an animal’s preference for dark locations and its urge to explore novel environments. Benzodiazapines and lesions of the ventral hippocampus have been shown to increase the amount of time animals spend in the white part of the black-white alley (Bannerman et al. 2003; McHugh et al. 2004).

The test apparatus consisted of a long alley (120 cm long × 9 cm wide × 29 cm high), with the floor and walls of one half painted black and the other half painted white. Each mouse was placed individually into the black section of the test alley, adjacent to, and facing, the end wall. The mouse was observed for 120 sec. Latency to first cross from the black section to the white section, total time spent in the white section, and number of crossings between the two sections were recorded. If the animals had not entered the white alley by the end of the test, they were assigned a latency of 120 sec.

### Locomotor Activity

Spontaneous locomotor activity was assessed in a novel environment. Mice were placed individually in transparent plastic cages (26 cm long; 16 cm wide; 17 cm high) with ventilated lids and a thin layer (0.5 cm) of bedding. Two infrared beams crossed each cage 1.5 cm above the floor, with each beam 7 cm from the centre of the cage. The number of beam breaks made per 5 minute time bin was recorded during a 2 hour test session.

### Species-Typical Behaviour

Species-typical behaviours are behaviours that are spontaneously emitted by a species in natural or semi-naturalistic settings. In mice these include behaviours such as nesting, burrowing, hoarding and digging (Dudek et al. 1983; Fantino and Cabanac 1984; Deacon 2006c, a, b).

#### Burrowing

In the wild, mice and other rodents often create burrows, which protect them from predators, allow them to store food, and provide a warm, sheltered environment for rearing young (Dudek et al. 1983). In the laboratory burrowing can be tested using a container filled with a substrate such as food pellets, earth, bedding or clay balls. The container is left in the home cage and the amount of burrowing can be determined by the quantity of substrate removed from the container (Deacon 2006b). Excitotoxic lesions of the hippocampus have been found to almost completely abolish burrowing (Deacon et al. 2002).

Burrows were made from 20 cm long (6.8 cm diameter) grey plastic cylinders raised at the open end by two 5 cm long screws. Each burrow was filled with 200 g of standard laboratory food pellets and placed against the long wall of a large cage. A mouse was placed in each cage and left undisturbed for 2 hours. The amount burrowed was recorded. Mice were provided with water throughout testing.

#### Marble Burying

Digging also represents a species-typical behaviour that can be measured in the laboratory (Deacon 2006c). Marble burying is impaired by benzodiazepines and SSRIs (at doses that do not affect locomotor activity) (Broekkamp et al. 1986; Njung'e and Handley 1991; Ichimaru et al. 1995) as well as hippocampal lesions (Deacon et al. 2002), suggesting that hippocampal circuits have a role in the expression of this behaviour.

Marble burying was tested with transparent plastic cages (26 cm long; 16 cm wide; 17 cm high) filled with a 10 cm deep layer of sawdust. 10 glass marbles (each approx 1.5 cm diameter) were placed in two rows on the top of the sawdust in each cage. A mouse was placed in each cage and left undisturbed for 30 minutes, after which the number of marbles that were buried to at least 2/3 of their depth was recorded.

Bannerman, D. M., Deacon, R. M., Offen, S., Friswell, J., Grubb, M. and Rawlins, J. N. (2002). "Double dissociation of function within the hippocampus: spatial memory and hyponeophagia." Behav Neurosci 116(5): 884-901.

Bannerman, D. M., Grubb, M., Deacon, R. M., Yee, B. K., Feldon, J. and Rawlins, J. N. (2003). "Ventral hippocampal lesions affect anxiety but not spatial learning." Behav Brain Res 139(1-2): 197-213.

Bengel, D., Murphy, D. L., Andrews, A. M., Wichems, C. H., Feltner, D., Heils, A., Mossner, R., Westphal, H. and Lesch, K. P. (1998). "Altered brain serotonin homeostasis and locomotor insensitivity to 3, 4-methylenedioxymethamphetamine ("Ecstasy") in serotonin transporter-deficient mice." Mol Pharmacol 53(4): 649-655.

Britton, D. R. and Britton, K. T. (1981). "A sensitive open field measure of anxiolytic drug activity." Pharmacol Biochem Behav 15(4): 577-582.

Broekkamp, C. L., Rijk, H. W., Joly-Gelouin, D. and Lloyd, K. L. (1986). "Major tranquillizers can be distinguished from minor tranquillizers on the basis of effects on marble burying and swim-induced grooming in mice." Eur J Pharmacol 126(3): 223-229.

Deacon, R. M. (2006a). "Assessing nest building in mice." Nat Protoc 1(3): 1117-1119.

Deacon, R. M. (2006b). "Burrowing in rodents: a sensitive method for detecting behavioral dysfunction." Nat Protoc 1(1): 118-121.

Deacon, R. M. (2006c). "Digging and marble burying in mice: simple methods for in vivo identification of biological impacts." Nat Protoc 1(1): 122-124.

Deacon, R. M., Croucher, A. and Rawlins, J. N. (2002). "Hippocampal cytotoxic lesion effects on species-typical behaviours in mice." Behav Brain Res 132(2): 203-213.

Dudek, B. C., Adams, N., Boice, R. and Abbott, M. E. (1983). "Genetic influences on digging behaviors in mice (Mus musculus) in laboratory and seminatural settings." J Comp Psychol 97(3): 249-259.

Fantino, M. and Cabanac, M. (1984). "Effect of a cold ambient temperature on the rat's food hoarding behavior." Physiol Behav 32(2): 183-190.

Handley, S. L. and Mithani, S. (1984). "Effects of alpha-adrenoceptor agonists and antagonists in a maze-exploration model of 'fear'-motivated behaviour." Naunyn Schmiedebergs Arch Pharmacol 327(1): 1-5.

Ichimaru, Y., Egawa, T. and Sawa, A. (1995). "5-HT1A-receptor subtype mediates the effect of fluvoxamine, a selective serotonin reuptake inhibitor, on marble-burying behavior in mice." Jpn J Pharmacol 68(1): 65-70.

Jennings, K. A., Loder, M. K., Sheward, W. J., Pei, Q., Deacon, R. M., Benson, M. A., Olverman, H. J., Hastie, N. D., Harmar, A. J., Shen, S. and Sharp, T. (2006). "Increased expression of the 5-HT transporter confers a low-anxiety phenotype linked to decreased 5-HT transmission." J Neurosci 26(35): 8955-8964.

Lister, R. G. (1987). "The use of a plus-maze to measure anxiety in the mouse." Psychopharmacology (Berl) 92(2): 180-185.

McHugh, S. B., Deacon, R. M., Rawlins, J. N. and Bannerman, D. M. (2004). "Amygdala and ventral hippocampus contribute differentially to mechanisms of fear and anxiety." Behav Neurosci 118(1): 63-78.

Merali, Z., Levac, C. and Anisman, H. (2003). "Validation of a simple, ethologically relevant paradigm for assessing anxiety in mice." Biol Psychiatry 54(5): 552-565.

Njung'e, K. and Handley, S. L. (1991). "Effects of 5-HT uptake inhibitors, agonists and antagonists on the burying of harmless objects by mice; a putative test for anxiolytic agents." Br J Pharmacol 104(1): 105-112.

Shen, S., Battersby, S., Weaver, M., Clark, E., Stephens, K. and Harmar, A. J. (2000). "Refined mapping of the human serotonin transporter (SLC6A4) gene within 17q11 adjacent to the CPD and NF1 genes." Eur J Hum Genet 8(1): 75-78.

Shephard, R. A. and Broadhurst, P. L. (1982). "Effects of diazepam and picrotoxin on hyponeophagia in rats." Neuropharmacology 21(8): 771-773.

Shephard, R. A., Jackson, H. F., Broadhurst, P. L. and Deakin, J. F. (1984). "Relationships between hyponeophagia, diazepam sensitivity and benzodiazepine receptor binding in eighteen rat genotypes." Pharmacol Biochem Behav 20(6): 845-847.
